# On the Influence of Structural Connectivity on the Correlation Patterns and Network Synchronization

**DOI:** 10.3389/fncom.2018.00105

**Published:** 2019-01-08

**Authors:** Parisa Sadat Nazemi, Yousef Jamali

**Affiliations:** Department of Mathematics, Tarbiat Modares University, Tehran, Iran

**Keywords:** correlation, synchronization, neural mass model, functional network, small-world network

## Abstract

Since brain structural connectivity is the foundation of its functionality, in order to understand brain abilities, studying the relation between structural and functional connectivity is essential. Several approaches have been applied to measure the role of the structural connectivity in the emergent correlation/synchronization patterns. In this study, we investigates the cross-correlation and synchronization sensitivity to coupling strength between neural regions for different topological networks. We model the neural populations by a neural mass model that express an oscillatory dynamic. The results highlight that coupling between neural ensembles leads to various cross-correlation patterns and local synchrony even on an ordered network. Moreover, as the network departs from an ordered organization to a small-world architecture, correlation patterns, and synchronization dynamics change. Interestingly, at a certain range of the synaptic strength, by fixing the structural conditions, different organized patterns are seen at the different input signals. This variety switches to a bifurcation region by increasing the synaptic strength. We show that topological variations is a major factor of synchronization behavior and lead to alterations in correlated local clusters. We found the coupling strength (between cortical areas) to be especially important at conversions of correlation and synchronization states. Since correlation patterns generate functional connections and transitions of functional connectivity have been related to cognitive operations, these diverse correlation patterns may be considered as different dynamical states corresponding to various cognitive tasks.

## Introduction

Brain, as a combination of neural ensembles, generates oscillatory activities. These oscillations can be recorded simultaneously from the neural masses with electroencephalography (EEG) and magnetoencephalography (MEG). As the neural populations generate oscillatory activities, we can model the brain dynamics as a system of coupled oscillators, and whenever a system of coupled oscillators is considered, synchronization will be the ubiquitous phenomenon.

Synchronization has been broadly analyzed at the level of individual neurons (Bazhenov et al., 2008; Bonjean et al., 2011; Feldt Muldoon et al., 2013). This phenomenon is slightly different in mesoscale. Synchronization in mesoscale defines as a correlated activity of two neural masses or the correlated spikes in two regions. Since the activity of each neural population is oscillatory, one can refer to neural populations as oscillators, which their phase defined as the state of each periodic oscillator. Phase synchronization indicates the dependency between oscillation phases in different masses or brain regions (Fell and Axmacher, 2011). In other words, when phases of oscillators in two regions become correlated, we can speak of phase synchronization. Two different types of synchronization are in-phase and anti-phase. In-phase synchronization defined as simultaneously firing patterns of neurons, and anti-phase is considered as an increase in the activity of a certain area of the brain, while the activity of others decreases (Li and Zhou, 2011).

In order to measure the synchronization of synaptic activity across distinct neural masses, it is possible to place multiple electrodes in different parts of the brain and record LFPs. The synchronized activity of the neural masses leads to large-amplitude oscillations of the LFP which can be recorded from outside of the scalp using EEG and MEG.

Analyzing EEG and MEG signals reveals that not only the oscillatory activity of distinct neural populations can synchronize, but also and more importantly behavioral or cognitive states depend on this synchronization and changes in correlation patterns of neural activity (Srinivasan et al., 1999; von Stein et al., 1999; Schnitzler and Gross, 2005).

The critical factor in synchronization is coupling. Connections between oscillators (units) transfer the activation state of oscillators to each other, and this causes alterations in the coupled oscillator phases. In order to quantify phase differences between neural populations, neuroscientists measure the correlation between pairs of neural masses activities. The first approaches to measuring the correlation between neurophysiological time series by recording simultaneously from two distinct anatomical locations were made more than 60 years ago (Brazier and Casby, 1952; Brazier and Barlow, 1956) and offer a very general time series analysis. The most classical way for time series analysis is to evaluate the correlation coefficient between the dynamical activities recorded from separate brain areas.

When we model a network of neural masses with a coupled oscillatory system, the correlation and synchronization between masses are nearly close concepts. Due to the fact that they both provided essentially the same information about the system of coupled oscillations (Mezeiová and Paluš, 2012). If two neural ensembles have a high correlation, this coherency will synchronize them. Besides, synchronized masses not only have a high correlation but also their phases are similar. For example, complete synchronization is always associated with high correlation between both amplitude and phase of two oscillators, which eventually lead oscillators to have identical states. Consequently, the correlation between neural populations measures the likelihood of their synchronization. However, we cannot use these two measures interchangeably. Owing to the fact that correlation and synchronization view the system coherency from different perspectives.

Furthermore, the correlation between brain regions besides other statistical dependencies indicates functional interactions known as “functional connectivity.” Simply put, functional connectivity between two locations is the existence of a correlation or synchronized dynamic activity (Friston, 1994). Many studies have been done to analyze functional connectivity in both micro-scale and mesoscale using synchronization and correlation measures (Ponten et al., 2009; Hlinka and Coombes, 2012; Stam et al., 2016).

Pearson correlation coefficient as the most widely used measure of functional connectivity examines the linear statistical dependence between variables. Functional connectivity can mainly be prescribed by correlation coefficient matrix which can be quantified by thresholding in order to define edges (with some thresholding techniques). Although in this paper we do not refer to the correlation matrix as functional connectivity, it is important to remember that correlation patterns describe activity at the functional level.

In this paper, our objective is to show how the topology of the network, i.e., structural connectivity (and coupling between units) can affect the correlation between units and the formation of synchronization patterns. For this purpose, we make use of a classical neural-mass model (Wilson and Cowan, 1972) and simulate neural dynamic in a way we could manipulate the coupling strength between areas. Thus, our primary goal is evaluating the sensitivity of correlation to the coupling strength. Using neural mass modeling, we assess network dynamics and measure the correlation coefficient between every two units. Correlation between units as a measure of statistical dependency represents functional connectivity. Here we are not interested in analyzing functional connectivity, instead, our focus is on studying synchronization behavior by considering different coupling coefficients and different topological networks.

The paper is organized as follows. First, we describe the theoretical framework which is used to simulate the dynamics of each structural unit of the neural system. Then we introduce different topological networks which are taken as structural connectivity. Later the simulation details are described and finally, we bring some achievements in this study and show the result of the simulation.

## MaterialS and Methods

### Wilson-Cowan Model

Dynamical models based on individual neurons' behavior are computationally inefficient for large-scale simulations. Some techniques have been proposed to reduce computational complexity, including employing neural mass models (Beim Graben and Rodrigues, 2012). These models apply mean-field approximations and describe the activity of the population as an average activity of all neurons within the population. Each neural masses or populations of neurons generate a mesoscopic unit, which serves as a node in large-scale brain networks (Gray and Robinson, 2013; Roy and Jirsa, 2013).

For simulation, we consider the Wilson-Cowan neurodynamic activity-based model for each node (Wilson and Cowan, 1972). The Wilson-Cowan model is one of the most influential models in computational neuroscience which describes the activity of each neural population as mean firing rate of its excitatory and inhibitory subpopulations by using the mean-field approximation and non-linear differential equations. These excitatory and inhibitory cells within each population are assumed to be in close spatial proximity with dense interconnections, so there would be a path between any two cells, which means that there wouldn't be any isolated cell within the population.

**Figure 1:**
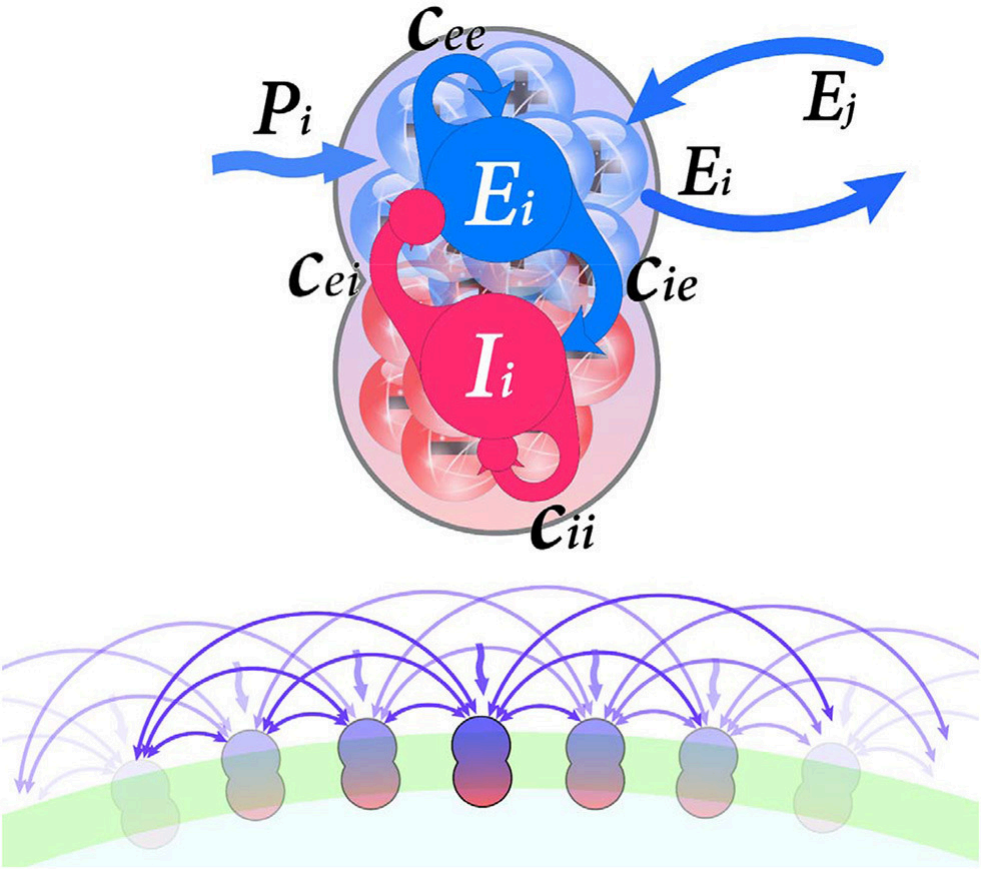
(Upper) Each neural population i contain excitatory and inhibitory subpopulations (E_i_ and I_i_, respectively) which are interconnected by synaptic weights *c*_*EE*_, *c*_*IE*_, *c*_*EI*_, *c*_*II*_. Nodes connected through their excitatory units. Furthermore, external input P_i_ stimulate excitatory units. (Lower) Schematic representation of the regular ring lattice network where each node is connected to six neighbors.

As indicated in Figure 1, each node consists of a population of excitatory neurons, which is coupled with a population of inhibitory neurons. Furthermore, network structure units are coupled through the excitatory units. The equations for the classical Wilson-Cowan model are as follows:

$$\begin{array}{l} \tau_{E}\frac{d}{dt}E_{k}=\;-E_{k}+S\left[a_{E}\left(c_{EE}E_{k}-c_{EI}I_{k}-\theta_{E}+P_{k}+\upsilon \overset{N}{\underset{l=1}{\sum }}C_{kl}E_{l}\right)\right]\;\\ \;\tau_{I}\frac{d}{dt}I_{k}=\;-I_{k}+S\left[a_{I}\left(c_{IE}E_{k}-c_{II}I_{k}-\theta_{I}\right)\right] \end{array}$$

Where, subscripts *E* and *I* indicate excitatory and inhibitory neurons, respectively. *E*_*k*_ is the average activity of the excitatory population of the *k*^*th*^ node, *I*_*k*_ is the average activity of *k*^*th*^ node inhibitory cells. The average activity can be defined as the portion of cells in the population which are firing per unit time. Excitatory population membrane time-constant is τ_*E*_ and inhibitory population, membrane time-constant is τ_*I*_. S is a sigmoid *response function* which transforms current into discharge rate, for each subpopulation with a specific input and time membrane constant.

$$S\left(x\right)=\;\frac{1}{1+\;e^{-x}}$$

The parameters *a* and θ are related to sigmoid function, *a* determines the position of the maximum slope, and θ is the value of the maximum slope of the sigmoid function, which are different for excitatory and inhibitory sub-populations.

External input *P*_*k*_, internal terms scaling by synaptic weights *c*_*EE*_, *c*_*IE*_, *c*_*EI*_, *c*_*II*_ and long-range coupling term $\nu \sum_{l=1}^{N}C_{kl}E_{l}$ are inputs of response function *S*. The parameter *P*_*k*_ is the activity coming from distant, known as the external perturbation or external stimulus to the excitatory sub-population, here present the input noise. Synaptic weights *c* represent coupling coefficients within each population (Figure 1). Long-range coupling term $\sum_{l=1}^{N}C_{kl}E_{l}$ is the input activity of neighbors which combines single units to form a network. This network represented by an adjacency matrix *C*_*kl*_ (here considered as structural connectivity) and delineate propagation of neural activity along long-range tracts (i.e., white matter tracts). The coupling term's aim is to transform the entering activity (from the rest of the network) to a proper form for the model equations. Finally, the whole long-range coupling term is scaled via the overall coupling coefficient ν. In other words, coupling coefficient ν determines connections strength between units, whereas synaptic weights scale connections within each unit. For the further mathematical description, please refer to Wilson and Cowan (1972), Borisyuk and Kirillov (1992), and Decker and Noonburg (2012).

### Parameter Choices

A primary feature of the Wilson-Cowan model is that it exhibits different dynamical behavior and stable equilibria (Wilson and Cowan, 1972; Cowan et al., 2016). Depending on the chosen parameter, dynamic behavior ranges from resting in steady states to limit cycle oscillations. In this simulation, the variables of interest are *E* and *I*, i.e., excitatory and inhibitory activities. The fixed parameters values are *a*_*E*_ = 0.8, *a*_*i*_ = 0.8, θ_*E*_ = 2.0, θ_*i*_ = 8.0, τ_*E*_ = 0.125, τ_*i*_ = 0.25 and set of synaptic weights *c*_*EE*_ = 8, *c*_*IE*_ = 8, *c*_*EI*_ = 16, *c*_*II*_ = 4, which are determined in a way that each isolated individual unit displays limit cycle oscillations. Also for each unit, there is an input noise *P*_*k*_ from Brownian noise sample. Mathematical and dynamical analysis of a single Wilson-Cowan unit have been done in detail by Wilson and Cowan (1972) and many others (Borisyuk and Kirillov, 1992; Ledoux and Brunel, 2011; Decker and Noonburg, 2012).

It is important to note that in the network, each unit can display many different dynamic patterns from limit cycles with a different range, frequency and central point to fixed stable point. This is due to the coupling of units and connectivity effects. Since by connecting Wilson-Cowan units, a number of parameters drastically increases, mathematical analysis of a network of Wilson-Cowan units needs elaborate computations. Besides non-linearity of differential equations makes arduous mathematical challenges. Thus, mathematical analysis is replaced by numerical methods (Latham et al., 2000; Maruyama et al., 2014; Neves and Monteiro, 2016).

The specific characteristic of this model is that it exhibits oscillatory behavior, individual nodes oscillate intrinsically, and synchronization of these oscillators leads to network oscillations.

### Cross-Correlation

Traditionally, cross-correlation is a useful method for comparing time series. By using cross-correlation, we will be able to quantify the similarity between time series recorded from distinct neural populations. In this study cross-correlation between brain areas (nodes) is quantified via the Pearson correlation coefficient (*r*).

Pearson correlation coefficient indicates a statistical association between two variables of interest by measuring their linear relation. Since Pearson correlation coefficient is based on the method of covariance, it is considered as the best method of measuring the relationship between two continuous variables. Despite the fact that there are some other methods for quantifying correlation (Levine et al., 2017), here we only use Pearson cross-correlation.

The values of cross-correlation (*r*) are between −1 and +1 indicating positive and negative association, respectively, and *r* = 0 refers to the uncorrelated state. Correlation matrix is a symmetric matrix derived from measuring pairwise correlation coefficient of all nodes activity time series.

As mentioned above, cross-correlation and synchronization are close concepts. However, they are not transposable. Cross-correlation measures liner dependency but synchronization evaluates non-linear relations. Moreover, correlation provides an information about the association between every two oscillators, while the synchronization (derived from Kuramoto order parameter) have generally used to quantify the coherency among a group of oscillators.

Correlation outcome of this simulation is represented in two forms, one is correlation matrix depicted in a heat map, and the second is mean correlation coefficient for all units. General features of correlation matrices are that they are symmetric and their diagonal elements are one.

Since our objective is studying the effects of coupling on the correlation between units, the simulation outcomes are *r*(ν). The resulting *r*(ν) values are computed after the system went to its steady state and averaged over all simulations.

### Networks

A structural network represented by a graph whose verdicts are neural populations and its edges are inter-population connections. Neural populations are collections of neurons with assumed close spatially distribution and with adequate size to valid mean-field approximation. The graph is represented by *C* = *C*_*kl*_; where *C*_*kl*_ = 1 if there is a connection between population k and population l, otherwise *C*_*kl*_ will be zero.

**Figure 2:**
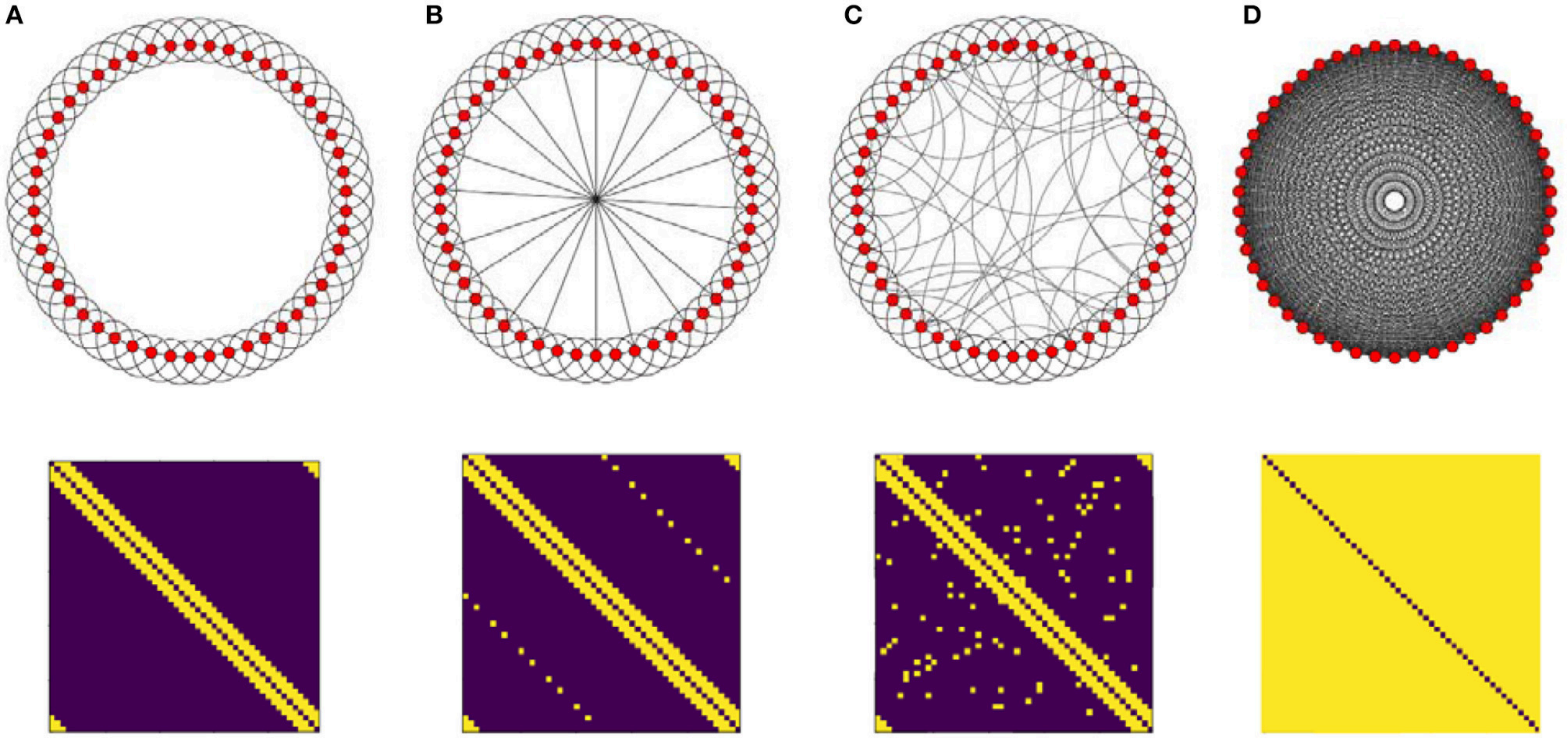
Illustration of the four topologies which are taken as structural connectivity architectures and their adjacency matrix (bottom). **(A)** Ring lattice network. **(B)** Ring lattice network after adding ten edges regularly. **(C)** Ring lattice network after adding 40 random edges. **(D)** Complete network.

In this research, three different connectivity topologies are taken as structural connectivity (*C*_*kl*_), regular, full connected and random. First, we start with a regular ring lattice network with 50 nodes where each one is connected to all of its six nearest neighbors. Another structural connectivity is induced by adding shortcuts to this ring. First, we add shortcuts in a regular way, then we add edges randomly. Note that adding regular shortcuts is different from random one. By adding random, the network exhibit small-world property which is similar to human brain organization but by adding regular one the system still remains regular but in the higher dimension. We also investigate fully connected network (Figure 2). Although the all-to-all network is far away from brain topological structure, it can exhibit the influence of increasing density of the graph on the activity patterns, so it is worth taking into account. In all cases, networks are undirected of size 50 and each node represents a population of excitatory and inhibitory neurons.

In the computational approach to modeling mesoscale neural networks, the spatial extent of neural populations is abstract, which can range from micro-columns to the whole brain (Sanz-Leon et al., 2015). In this research, we refer to neural populations as brain regions and assume that 50 of it can cover a whole cortical area.

### Simulation

As mentioned above, the excitatory unit's activity is the key variable, and it represents the whole unit activity. For analyzing effects of network topology on the activity and correlated activities, we simulate for different connectivity strengths so the outcome of simulation would be mean of network activity, *E*(ν), and mean of the correlation coefficient, *r*(ν). Furthermore, the spectrum of the network activity (*E*(ν)) is analyzed using Fast Fourier transformation techniques.

For each coupling strength coefficient ν, simulation is repeated 90 times and resulting *E*(ν) and *r*(ν) values are computed by averaging over all simulations.

## Results

### Ordered Network

We begin by considering the case in which network has a ring lattice structure and every unit is coupled to its six nearest neighbors.

**Figure 3:**
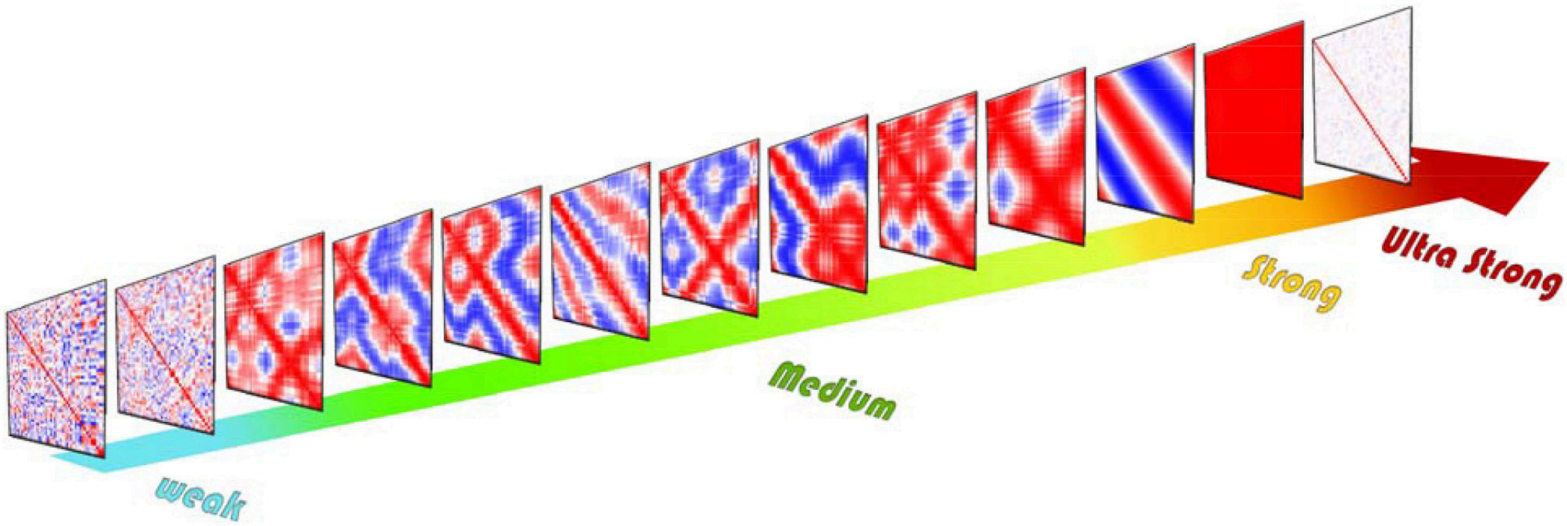
Schematic of different cross-correlation matrices across the coupling coefficient growth of the ordered network. Cross-correlation values range from +1 (red) to −1 (blue). For weak couplings, the resulting cross-correlation matrices are nearly random, but for the medium level of couplings in each repeat of simulation correlation exhibit completely different patterns. These various patterns show different correlated and synchronized clusters. For the strong level of couplings the system exhibit two-phase behavior which by repeating simulation cross-correlation matrices exhibit only two patterns of synchronization. If we increase coupling further, each unit leaves the limit cycle and rests in a fixed point. Thus, the correlation between units vanishes.

Coupling coefficient has a significant effect on the correlation between units. As shown in Figure 3 various correlation patterns are induced by changing the coupling coefficient. Based on the resulting correlation behavior, we categorized coupling ν in four intervals: weak, medium, strong, and ultra-strong.

**Figure 4:**
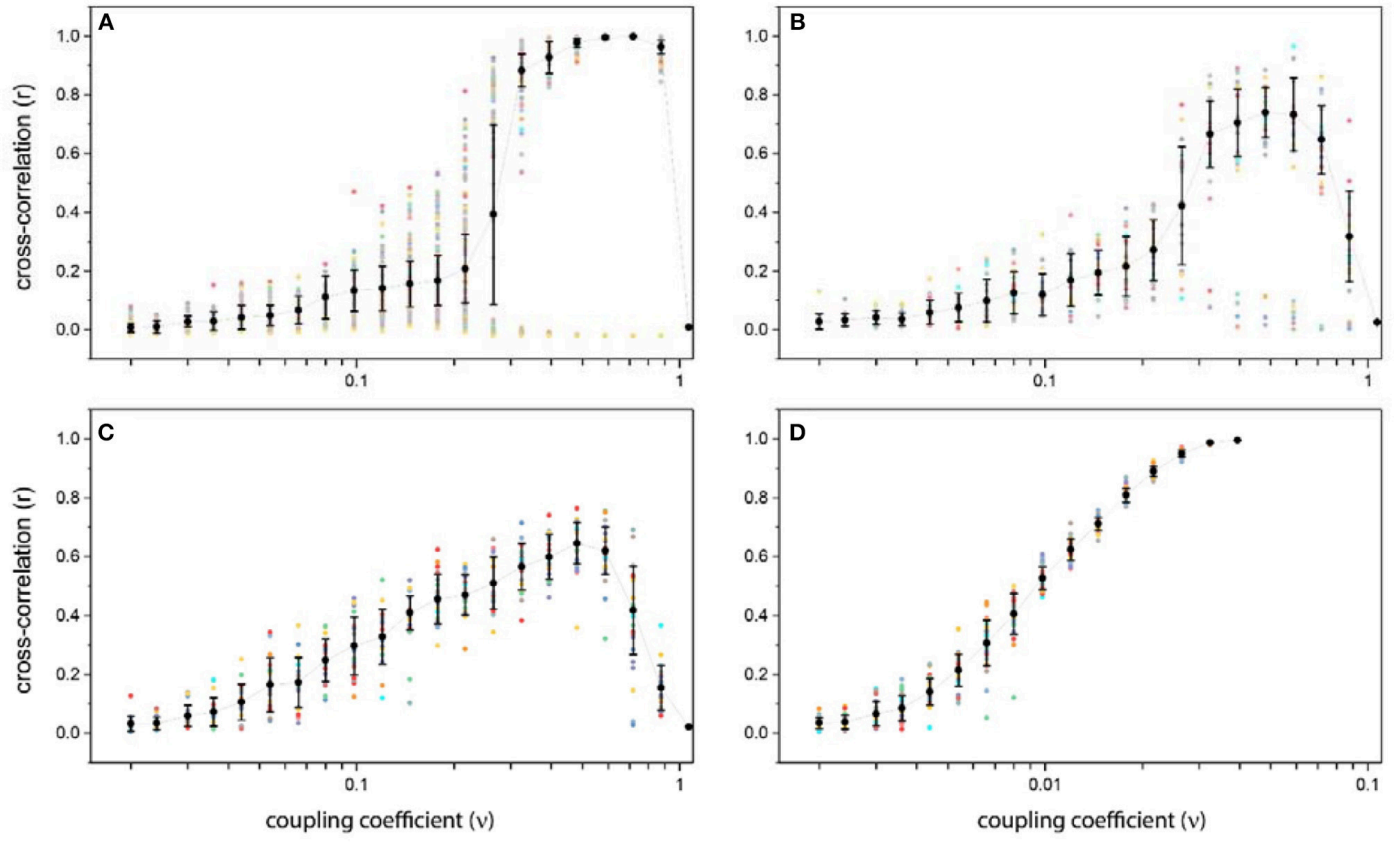
Average and variance of cross-correlation as a function of the coupling coefficient for different structural connectivity: **(A)** Ring lattice network. **(B)** Ring lattice network after adding ten edges regularly. **(C)** Ring lattice network after adding 40 random edges. **(D)** Complete network. The x-axis in all subplots express coupling coefficient ν and the y-axis is average cross-correlation. Each dot represents different realizations of the system.

Obviously, for weak couplings, oscillators will not have a significant effect on each other, so the correlation between units is low too. In this case, the resulting correlation matrices in all repetition are nearly the same, low positive and negative correlations occur randomly between units, and there is no specific pattern in those, so mean and variance of correlation coefficients are low too (Figure 4A). In this case, the system is not synchronized globally nor locally.

As we increase ν, correlation behavior changes drastically. Surprisingly, for coupling coefficients in a particular interval, correlation matrix shows different patterns as we repeat the simulation for specific coupling coefficient, i.e., in this range the resulting correlation matrix in each repetition is totally different from others. Consequently, the average correlation can take different values and variance of them is high (Figure 4A).

For most correlation matrices the uncorrelated state rarely happens. Instead, units had high positive or high negative correlations between themselves, which means that the system can generate some synchronized clusters, i.e., local synchrony. In high positive correlation areas, we have complete in-phase local synchronization, and in high negative correlation areas, we have complete anti-phase local synchronization. Furthermore, this synchronization of local clusters could not lead to global synchronization. The scale of this local synchrony varies from 4 masses to half of the network.

It is important to note that, in each repeat (run) due to different initial states *E*_0_, *I*_0_, and the existence of Brownian random input *P*_*k*_, the result is dissimilar from another repeat, which shows the sensivity of the system to its initial conditions. In mathematics and physics, this phenomenon is called chaotic behavior.

If we increase ν further, this chaotic behavior switches to another regime. “Strong” level of coupling between network units causes the system to have “two-phase” behavior. For ν in this level, correlation matrices for all repetitions show only two patterns. Which means that, there is just two main attractors and starting from each random initial state, the system would end up in one of these two phases. In fact, for strong couplings, a symmetry breaking occurs. One shows a high positive correlation between all units, which lead the global synchronization in the system. And the other shows high positive correlation for structurally connected nodes and their near neighbors along with high negative correlation for structurally unconnected nodes (Figure 4A). Because of this symmetry breaking, in some repetitions, we have complete global synchronization and in others the system exhibit local synchrony within half of the network populations.

For higher couplings in “ultra-strong” level, individual oscillators leave the limit cycle regime, relaxed in a steady state and stop oscillating, due to excessive input. In this situation, their activity time series will not exhibit any oscillation so there wouldn't be any correlation between nodes and all entries of cross-correlation matrix will be near zero indicating an uncorrelated phase and no synchrony (Figure 4A).

The videos in the Supplementary Material show the evolution of node dynamics and correlation coefficient during the time, the samples are taken every 200 steps, and correlation matrix in each snapshot is calculated for the last 200 steps.

**Figure 5:**
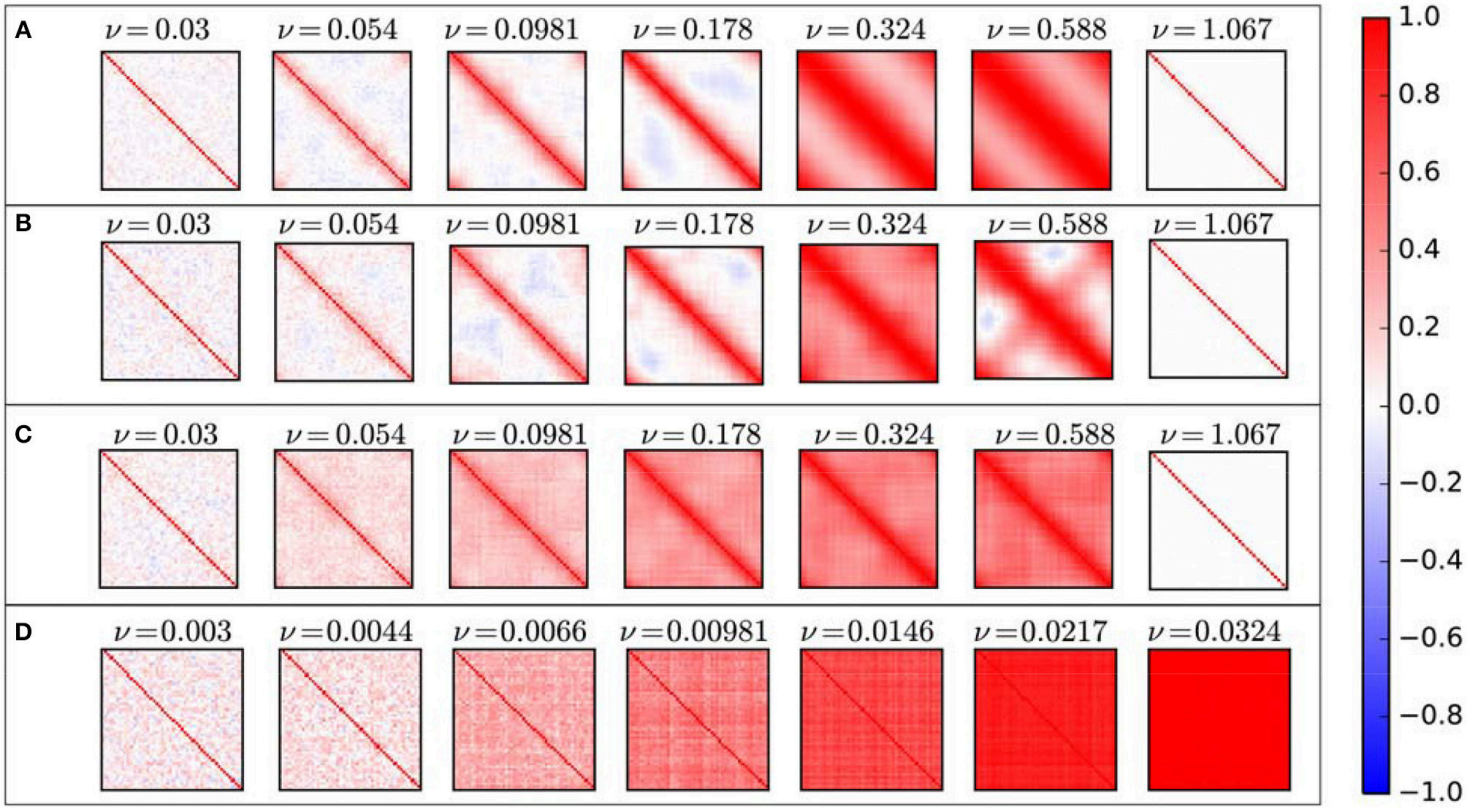
Average cross-correlation matrices for different structural connectivity: **(A)** Ring lattice network. **(B)** Ring lattice network after adding ten edges regularly. **(C)** Ring lattice network after adding 40 random edges. **(D)** Complete network.

As mentioned above, for some couplings, by repeating simulation, correlation coefficient matrix exhibit completely different patterns (Figure 3). But how is its overall behavior? In Figure 5 we can see the average correlation coefficient matrices over 90 repetitions for some specific coupling coefficients ν.

For low couplings, on average, directly connected nodes have a positive correlation between each other, and other nodes are not correlated or have a negative correlation. By increasing coupling ν, besides directly connected nodes, we will have a positive correlation between each node and its k-nearest neighbors. This k is increasing of coupling ν. As ν increases, each node correlates positively with its k-step near neighbors and negatively or it is uncorrelated with other nodes, i.e., positive correlation occur between node within clusters and others are uncorrelated or have a slightly negative correlation. As mentioned above, for the medium level of ν, correlation matrix shows various patterns. Here average matrices do not exhibit those patterns, they only continue the previous process where nodes are positively correlated with more near neighbors and are uncorrelated with nodes in the farthest side of the ring network. Importantly there is no significant negative correlation at all. Furthermore, for ultra-strong couplings, the average correlation matrix indicates zero value between all nodes where there isn't any correlation between units as they don't oscillate anymore.

**Figure 6:**
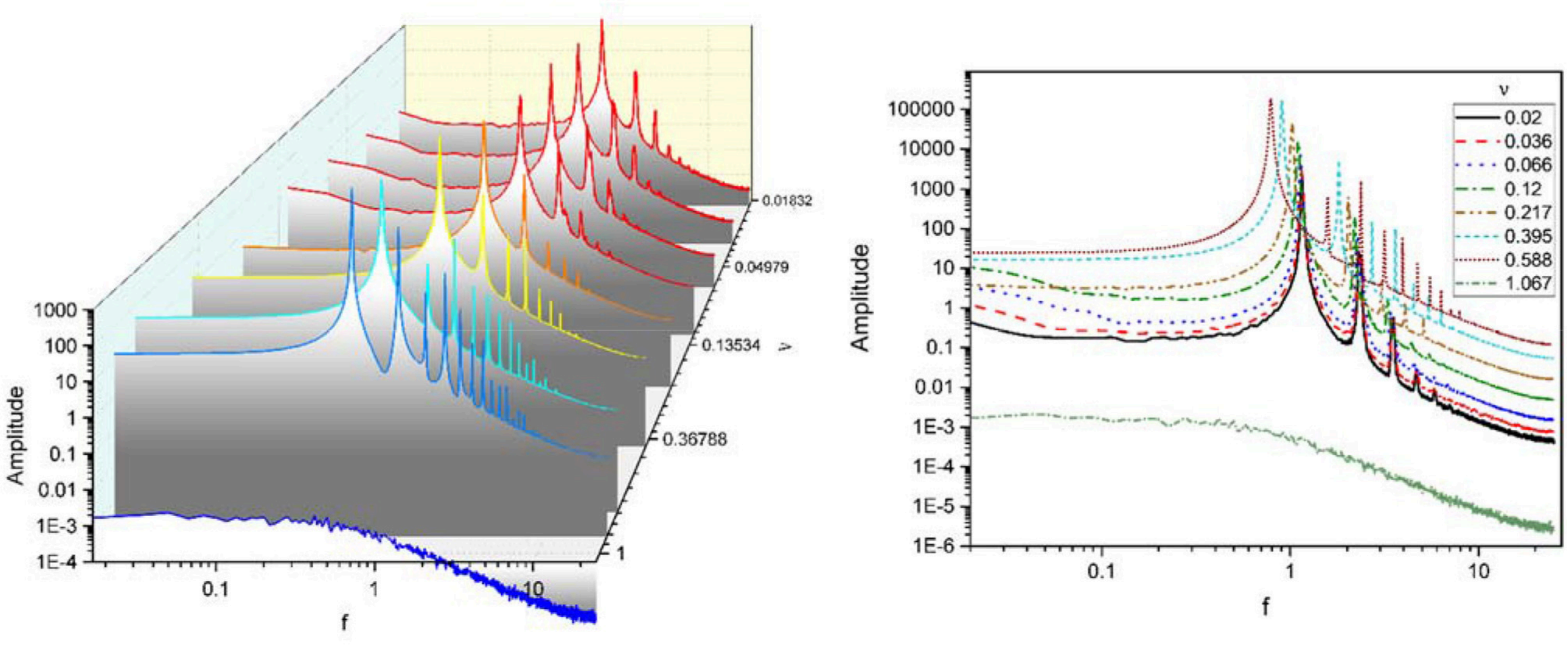
Power spectra for the mean activity of excitatory populations for different coupling coefficients ν. **(Left)** Power spectra are plotted separately. **(Right)** Power spectra overlay. Although it is not a normalized form of the power spectrum, it is still showing that oscillations main power occur in delta-band.

We have also investigated the power spectrum of data using fast Fourier transform (FFT) which is shown in Figure 6. On the left, power spectrums for different couplings are plotted separately, and on the right, power spectra overlay. In fact, both plots express one concept. It must be noted that this power spectrum is not normalized.

The power spectra of mean excitatory activities for different coupling strengths in the Figure 6 indicates a significant difference between ultra-strong and other weaker couplings.

Below a critical coupling ν, the spectrum of excitatory activity for each coupling coefficient has a major peak (main power) in a specific frequency near 1 Hz, in the delta band, and power-law decay with the same exponent near 2. Increasing coupling until critical ν, moves peak of the power spectrum to the left while provoking a dramatic power growth, i.e., as the coupling between units becomes stronger, the frequency of activity oscillations decline while their amplitude rise. Weak couplings lead to high frequency and low amplitude oscillations, but strong couplings induce low frequency with high amplitude oscillations. In other words, in this case, as coupling increased the power peak shifted to lower frequencies but oscillations had higher power (high energy). This phenomena happened as a result of increasing coupling strength between units. For weak coupling, each unit produce weak oscillations with high frequencies, which means that the oscillation of each unit has small amplitude and fluctuates rapidly. But when the impact of units on each other increase system shows synchronization and equivalently units act similarly and produce oscillations with low frequency and big amplitude.

Increasing ν again after this critical value lead to a significantly different behavior. For ultra-strong couplings, there is no peak and the spectrum is close to flat before starting a power-law decay, as for this couplings each unit leaves limit cycle and stop oscillating.

### Ordered Network With Regular Shortcuts

In this part, structural connectivity, *C*_*kl*_, is changed by adding 10, 20, 30, 40, and 50 edges as shortcuts orderly, i.e., not randomly, by each shortcut, a couple of nodes with the longest distance are connected. In all cases, the resulting networks still have a regular organization.

Since the mean correlation behavior does not change significantly by adding regular shortcuts, the results for only one case have been mentioned. The average and variance of correlation coefficients as a function of coupling ν for a 10 number of shortcuts is shown in Figure 4B.

In all cases before the two-phase regime mean correlation is an increasing function of coupling. Furthermore, for all number of shortcuts this increase obey power-law function with roughly the same exponent and patterns of correlation have not changed significantly by adding these shortcuts to the network. Similarly, average correlation matrices are like ring lattice networks (Figure 5B).

In summary, adding regular shortcuts does not change the overall behavior of our system. Maybe because adding regular shortcuts does not change the ring lattice network topologically, notably it does not decline path length.

### Ordered Network With Random Shortcuts

Empirical networks are unlikely to have an ordered structure. Recent studies indicate that, human brain structural network along with C. Elegans (Watts and Strogatz, 1998), mouse brain network (Oh et al., 2014; Rubinov et al., 2015), cat and macaque (visual) cortex (Hilgetag et al., 2000; Hilgetag and Kaiser, 2004) have small-world architecture (Chen et al., 2008; Hagmann et al., 2008; van den Heuvel and Sporns, 2011). Small-world topology facilitates information segregation and integration which are essential for brain function (Liao et al., 2017).

Starting from a ring lattice network where nodes are connected to six near neighbors, then add new edges randomly. These new random edges are like shortcuts which connect distant units and reduce path length dramatically. In this paper, we add 5, 10, 20, 30, 40, 50, and 60 shortcuts. By adding these random shortcuts, the network does not have a regular organization anymore, instead shows a small-world architecture.

In comparison to the ordered network, here the system showed more varied patterns of correlation for the medium level of couplings, but these correlations are not high mostly. Which means that the system can be locally synchronized but not globally, and more importantly the system does not have a complete synchronization. Figure 4C shows that after adding forty shortcuts to the network, no bifurcation occurs.

**Figure 7:**
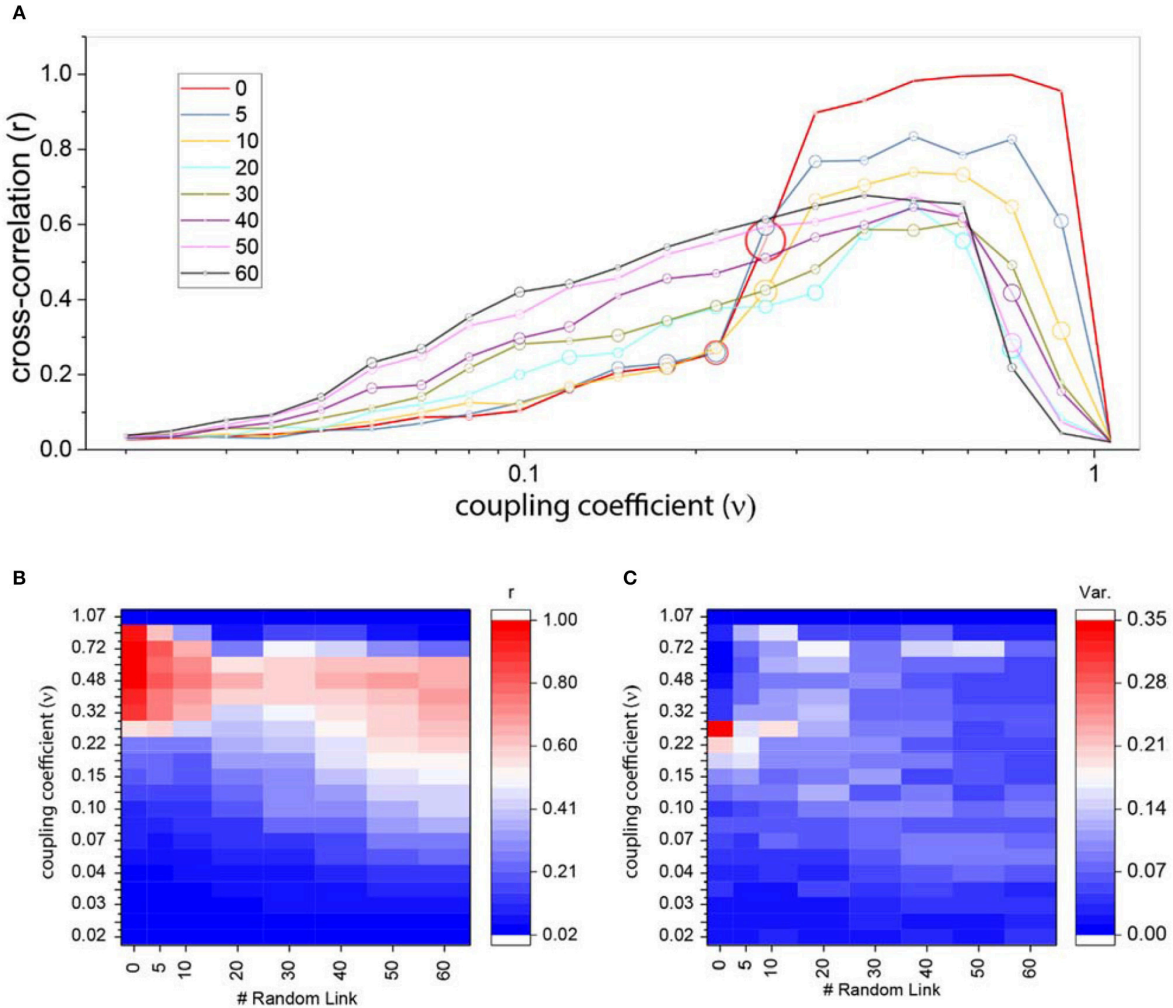
Cross-correlation for ring lattice network with random edges. **(A)** Average cross-correlation coefficient for a different number of random edges. The radius of circles represents the variance of the results. For better comparison, we also plot the average correlation of regular ring lattice network (red line). **(B)** An alternative representation of part **(A)** results. Average cross-correlation heat-map where the x-axis is the number of new random edges and y-axis is coupling coefficients. **(C)** The variance of cross-correlations heat-maps where axis are same as part **(B)**.

The average cross-correlation in Figure 7A shows an interesting difference between ordered and random ring lattice. If we look closer to high couplings level, we will notice the effects of random edges. In strong couplings, correlation of the ring lattice without any shortcut (red line) reaches the maximum of one, but as we attach more shortcuts to the network the correlation strength declines, i.e., maximum correlation reduces as the number of shortcuts increase. The strength of correlation for a different number of links and coupling coefficients in Figure 7B indicates this wane.

Although correlation patterns are diverse, they don't exhibit high positive and high negative cross-correlations. In fact, negative cross-correlations infrequently occur in comparison to an ordered network. Thus, the variance of the results for different couplings and a different number of edges is almost very low (Figure 7C).

As we mentioned above, the correlation becomes zero when oscillators leave limit cycle. Figure 7A shows that by adding more edges, this phenomenon happens in the lower couplings. The brisk decrease shifts to the left as edges attached to the ordered network. Furthermore, the increase in correlation from weak couplings to medium and high couplings happens more smoothly, despite the ordered network where correlation rise sharply to its maximum level.

Moreover, in comparison to the ordered ring lattice network, random shortcuts lead the average cross-correlation matrices to have a less negative correlation (Figure 5C). Consequently, anti-phase synchrony rarely is observed.

### Complete Network

We also investigate the correlation behavior in the complete network (Figure 4D). In this case, we deal with a dense network, so the impact of the density of the topological structure on the activity and correlation state can be seen.

According Figure 4D for the medium level of couplings, the variance of the cross-correlation states is small so the complete network does not exhibit any significant dissimilar correlation pattern, despite ring lattice network. Furthermore, there isn't any two-phase behavior for strong couplings.

It is important to note that, complete network receive inputs in higher order. For example, each unit in ring lattice network receives inputs from its six neighbors, but for complete network, units' inputs come from frothy nine other units. Consequently, complete network exhibits high correlation and global synchronization for lower orders of couplings in comparison to our previous networks.

In general, cross-correlation matrices do not exhibit any significant negative correlation between neural masses. Thus, maximum correlation achieves for lower couplings. So as coupling increases, correlation matrix without exhibiting negative correlations goes to the full correlated phase smoothly, where all of the neural masses have a positive correlation and complete synchronization (Figure 5D).

## Discussion

A network of neural masses can display synchronized patterns. The network architecture is one of the critical factors which determine ranges of this synchrony if it is local or global, in-phase or anti-phase, strong or weak. In this study, a neural mass model is utilized to investigate the overall network behavior at the mesoscopic scale. We investigated the effect of topological structure on correlation and synchronization between neural populations.

Our results highlight that, even on an ordered network, correlation is sensitive not only to coupling but also to initial state and external noise. For weak and ultra-strong couplings, this sensitivity diminishes, but for medium and strong couplings this sensitivity is maximum so we can see different correlation patterns and two-phase behavior, respectively. This chaotic behavior happens because of two reasons: First, for only a specific range of coupling coefficient, the system becomes extremely sensitive to initial conditions, which suggests that the coupling strength is crucial to this chaos. Second, the topological structure of the network forces the system to form different correlated clusters and produce local synchrony, since the clustering coefficient of the ring lattice network is high. We omit technical analysis of chaos in our system and encourage readers to look at (Alligood et al., 1997; Sprott, 2003) for further studies.

For medium and strong couplings the network can generate local synchronization in clusters and global in-phase synchrony. This in-cluster (local) synchronization leads to more local functional activity and thus more modular functional connectivity. Furthermore, the frequency distribution of oscillators slightly shifted toward lower frequencies with high amplitude as the coupling between them become stranger. Oscillations occurred mainly in beta-band.

As the network structure departs from ordered architecture and exhibits small-world organization, two-phase behavior vanishes and the network exhibit more harmonic oscillations. So we can predict that, for the human brain with small-world modular structure, the two-phase behavior is unlikely. Furthermore, due to structural complexity, more variant correlation and synchronization states emerged.

The clustering coefficient of the ring lattice network is higher that small-world topology. In ordered lattice network high correlated clusters are created. While in the small-world network, the strength of correlation between clusters diminishes compared to ordered lattice network. Therefore, it is safe to deduce that higher clustering coefficients in structural connectivity results in a stronger correlation between clusters.

Results from all-to-all network indicate that in a high densely network negative correlation and uncorrelated state are unlikely, in contrast to regular and small-world networks. Thus, we can predict that by increasing the density of the structural network, the state of correlation and synchronization among neural population shifts to high correlated masses and complete synchronization. One can conclude that correlation and synchronization are depend on two properties of the topological structure of the network: coupling coefficient among neural assemblies and density of the network.

We also computed synchronization via phase locking index (Kuramoto order parameter) (Kuramoto, 1975, 1984) and found (result not shown) that for each topological structure the overall synchronization behavior was the same as the average correlation coefficient, showing that correlation and synchronization are two sides of the same coin.

Cognitive performances are thought to emerge from the dynamic changes of functional connectivity and functional networks obtained from the correlation between anatomically distributed yet connected cortical areas (Fornito et al., 2012; Braun et al., 2015; Liang et al., 2016). Consequently, studying correlation patterns demonstrate the brain performance to meet its cognitive demands.

Variations in brain functionality have been related to cognitive operations. In this study, we indicated a link between brain structural connectivity and its functional organization. We showed that both coupling strength (topological variations) and external noise are significant factors of synchronization behavior and lead to alterations in correlated local clusters. We found the coupling strength to be especially important at conversions of correlation and synchronization states. The results suggest that each of the various correlation patterns in this paper can be taken as an attractor and may express dynamic changes of neural activities for a cognitive state.

## Author Contributions

YJ designed the study, supervised the project, and contributed to the final version of the manuscript. PN wrote the manuscript with the advice of YJ. All authors contributed to the simulation and discussed the results. PN and YJ devised the project, the main simulation ideas.

### Conflict of Interest Statement

The authors declare that the research was conducted in the absence of any commercial or financial relationships that could be construed as a potential conflict of interest.

## References

[B1] Alligood K. Sauer T. D. Yorke J. A. (1997). Chaos: An Introduction to Dynamical Systems. New York, NY: Springer.

[B2] Bazhenov M. Rulkov N. F. Timofeev I. (2008). Effect of synaptic connectivity on long-range synchronization of fast cortical oscillations. J. Neurophysiol. 100, 1562–1575. 10.1152/jn.90613.2008 18632897 PMC2652170

[B3] Beim Graben P. Rodrigues S. (2012). A biophysical observation model for field potentials of networks of leaky integrate-and-fire neurons. Front. Comput. Neurosci. 6:100. 10.3389/fncom.2012.00100 23316157 PMC3539751

[B4] Bonjean M. Baker T. Lemieux M. Timofeev I. Sejnowski T. Bazhenov M. (2011). Corticothalamic feedback controls sleep spindle duration *in vivo*. J. Neurosci. 31, 9124–9134. 10.1523/JNEUROSCI.0077-11.2011 21697364 PMC3131502

[B5] Borisyuk R. M. Kirillov A. B. (1992). Bifurcation analysis of a neural network model. Biol. Cybern. 66, 319–325. 10.1007/BF00203668 1550881

[B6] Braun U. Schäfer A. Walter H. Erk S. Romanczuk-Seiferth N. Haddad L.. (2015). Dynamic reconfiguration of frontal brain networks during executive cognition in humans. Proc. Nat. Acad. Sci. U.S.A. 112, 11678–11683. 10.1073/pnas.1422487112 26324898 PMC4577153

[B7] Brazier M. A. Barlow J. S. (1956). Some applications of correlation analysis to clinical problems in electroencephalography. Electroencephalogr. Clin. Neurophysiol 8, 325–331. 10.1016/0013-4694(56)90124-9 13317822

[B8] Brazier M. A. Casby J. U. (1952). Cross-correlation and autocorrelation studies of electroencephalographic potentials. Electroencephalogr. Clin. Neurophysiol. Suppl. 4, 201–211. 10.1016/0013-4694(52)90010-2 13033798

[B9] Chen Z. J. He Y. Rosa-Neto P. Germann J. Evans A. C. (2008). Revealing modular architecture of human brain structural networks by using cortical thickness from MRI. Cereb. Cortex 18, 2374–2381. 10.1093/cercor/bhn003 18267952 PMC2733312

[B10] Cowan J. D. Neuman J. van Drongelen W. (2016). Wilson–Cowan equations for neocortical dynamics. J. Math. Neurosci. 6:1. 10.1186/s13408-015-0034-5 26728012 PMC4733815

[B11] Decker R. Noonburg V. (2012). A periodically forced Wilson–Cowan system with multiple attractors. SIAM J. Math. Anal. 44, 887–905. 10.1137/110823365

[B12] Feldt Muldoon S. Soltesz I. Cossart R. (2013). Spatially clustered neuronal assemblies comprise the microstructure of synchrony in chronically epileptic networks. Proc. Nat. Acad. Sci. U.S.A. 110, 3567–3572. 10.1073/pnas.1216958110 23401510 PMC3587208

[B13] Fell J. Axmacher N. (2011). The role of phase synchronization in memory processes. Nat. Rev. Neurosci. 12:105. 10.1038/nrn2979 21248789

[B14] Fornito A. Harrison B. J. Zalesky A. Simons J. S. (2012). Competitive and cooperative dynamics of large-scale brain functional networks supporting recollection. Proc. Nat. Acad. Sci. U.S.A. 109, 12788–12793. 10.1073/pnas.1204185109 22807481 PMC3412011

[B15] Friston K. J. (1994). Functional an effective connectivity in neuroimaging: a synthesis. Hum. Brain Mapp. 2, 56–78. 10.1002/hbm.460020107

[B16] Gray R. T. Robinson P. A. (2013). Stability constraints on large-scale structural brain networks. Front. Comput. Neurosci. 7:31. 10.3389/fncom.2013.00031 23630490 PMC3624092

[B17] Hagmann P. Cammoun L. Gigandet X. Meuli R. Honey C. J. Wedeen V. J.. (2008). Mapping the structural core of human cerebral cortex. PLoS Biol. 6:e159. 10.1371/journal.pbio.0060159 18597554 PMC2443193

[B18] Hilgetag C. C. Burns G. A. O'Neill M. A. Scannell J. W. Young M. P. (2000). Anatomical connectivity defines the organization of clusters of cortical areas in the macaque monkey and the cat. Philos. Transac. Roy. Soc. B Biol. Sci. 355, 91–110. 10.1098/rstb.2000.0551 10703046 PMC1692723

[B19] Hilgetag C. C. Kaiser M. (2004). Clustered organization of cortical connectivity. Neuroinformatics 2, 353–360. 10.1385/NI:2:3:353 15365196

[B20] Hlinka J. Coombes S. (2012). Using computational models to relate structural and functional brain connectivity. Eur. J. Neurosci. 36, 2137–2145. 10.1111/j.1460-9568.2012.08081.x 22805059 PMC3437497

[B21] Kuramoto Y. (1984). Chemical Oscillations, Waves, and Turbulence. New York, NY: Springer-Verlag.

[B22] Kuramoto Y. H. A. (ed.). (1975). Lecture notes in physics, in International Symposium on Mathematical Problems in Theoretical Physics (Kyoto: Springer).

[B23] Latham P. E. Richmond B. J. Nelson P. G. Nirenberg S. (2000). Intrinsic dynamics in neuronal networks. I. Theory. J. Neurophysiol. 83, 808–827. 10.1152/jn.2000.83.2.808 10669496

[B24] Ledoux E. Brunel N. (2011). Dynamics of networks of excitatory and inhibitory neurons in response to time-dependent inputs. Front. Comput. Neurosci. 5:25. 10.3389/fncom.2011.00025 21647353 PMC3103906

[B25] Levine M. E. Langfelder P. Horvath S. (2017). A weighted SNP correlation network method for estimating polygenic risk scores. Methods Mol. Biol. 1613, 277–290. 10.1007/978-1-4939-7027-8_10 28849564 PMC5998804

[B26] Li D. Zhou C. (2011). Organization of anti-phase synchronization pattern in neural networks: what are the key factors? Front. Syst. Neurosci. 5:100. 10.3389/fnsys.2011.00100 22232576 PMC3233683

[B27] Liang X. Zou Q. He Y. Yang Y. (2016). Topologically reorganized connectivity architecture of default-mode, executive-control, and salience networks across working memory task loads. Cereb. Cortex 26, 1501–1511. 10.1093/cercor/bhu316 25596593 PMC4785946

[B28] Liao X. Vasilakos A. V. He Y. (2017). Small-world human brain networks: perspectives and challenges. Neurosci. Biobehav. Rev. 77, 286–300. 10.1016/j.neubiorev.2017.03.018 28389343

[B29] Maruyama Y. Kakimoto Y. Araki O. (2014). Analysis of chaotic oscillations induced in two coupled Wilson–Cowan models. Biol. Cybern. 108, 355–363. 10.1007/s00422-014-0604-8 24789794

[B30] Mezeiová K. Paluš M. (2012). Comparison of coherence and phase synchronization of the human sleep electroencephalogram. Clin. Neurophysiol. 123, 1821–1830. 10.1016/j.clinph.2012.01.016 22361266

[B31] Neves L. L. Monteiro L. H. A. (2016). A linear analysis of coupled Wilson-Cowan neuronal populations. Comput. Intellig. Neurosci. 2016:6. 10.1155/2016/8939218 27725829 PMC5048090

[B32] Oh S. W. Harris J. A. Ng L. Winslow B. Cain N. Mihalas S.. (2014). A mesoscale connectome of the mouse brain. Nature 508:207. 10.1038/nature13186 24695228 PMC5102064

[B33] Ponten S. C. Daffertshofer A. Hillebrand A. Stam C. J. (2009). The relationship between structural and functional connectivity: graph theoretical analysis of an EEG neural mass model. Neuroimage 52, 985–994. 10.1016/j.neuroimage.2009.10.049 19853665

[B34] Roy D. Jirsa V. (2013). Inferring network properties of cortical neurons with synaptic coupling and parameter dispersion. Front. Comput. Neurosci. 7:20. 10.3389/fncom.2013.00020 23533147 PMC3607799

[B35] Rubinov M. Ypma R. J. F. Watson C. Bullmore E. T. (2015). Wiring cost and topological participation of the mouse brain connectome. Proc. Nat. Acad. Sci. U.S.A. 112, 10032–10037. 10.1073/pnas.1420315112 26216962 PMC4538676

[B36] Sanz-Leon P. Knock S. A. Spiegler A. Jirsa V. K. (2015). Mathematical framework for large-scale brain network modeling in The Virtual Brain. Neuroimage 111, 385–430. 10.1016/j.neuroimage.2015.01.002 25592995

[B37] Schnitzler A. Gross J. (2005). Normal and pathological oscillatory communication in the brain. Nat. Rev. Neurosci. 6, 285–296. 10.1038/nrn1650 15803160

[B38] Sprott J. C. (2003). Chaos and Time Series Analysis. New York, NY: Oxford University Press.

[B39] Srinivasan R. Russell D. P. Edelman G. M. Tononi G. (1999). Increased synchronization of neuromagnetic responses during conscious perception. J. Neurosci. 19, 5435–5448. 10.1523/JNEUROSCI.19-13-05435.1999 10377353 PMC6782339

[B40] Stam C. J. van Straaten E. C. W. Van Dellen E. Tewarie P. Gong G. Hillebrand A.. (2016). The relation between structural and functional connectivity patterns in complex brain networks. Int. J. Psychophysiol. 103, 149–160. 10.1016/j.ijpsycho.2015.02.011 25678023

[B41] van den Heuvel M. P. Sporns O. (2011). Rich-club organization of the human connectome. J. Neurosci. 31, 15775–15786. 10.1523/JNEUROSCI.3539-11.2011 22049421 PMC6623027

[B42] von Stein A. Rappelsberger P. Sarnthein J. Petsche H. (1999). Synchronization between temporal and parietal cortex during multimodal object processing in man. Cereb. Cortex 9, 137–150. 10.1093/cercor/9.2.137 10220226

[B43] Watts D. J. Strogatz S. H. (1998). Collective dynamics of ‘small-world’ networks. Nature 393:440. 9623998 10.1038/30918

[B44] Wilson H. R. Cowan J. D. (1972). Excitatory and inhibitory interactions in localized populations of model neurons. Biophys. J. 12:24. 10.1016/S0006-3495(72)86068-5 4332108 PMC1484078

